# Developing a European internet and kiosk-based health information system

**DOI:** 10.2196/jmir.3.1.e6

**Published:** 2001-03-17

**Authors:** Adrian Moore, Gerard Parr, Mark Logan, Hayley Neely, Dietmar Roesner, Uew Dürer

**Affiliations:** ^1^University of Ulster at ColeraineNorthern Ireland; ^2^Otto-von-Guericke-UniversitätMagdeburgGermany

**Keywords:** Telemedicine, Internet, Kiosk, Health information system, Health promotion, Metatags, XML, Ontology

## Abstract

A consortium of partner organisations (universities, health care organisations and information technology companies) from Northern Ireland, Germany, Portugal and Italy have collaborated to develop a multi-lingual, multi-media Internet and kiosk-based health information system in cardiology and skin cancer.

The project, CATCH II (Citizens Advisory System based on Telematics for Communication and Health), has been funded by the European Commission under the Fourth Framework Research and Development TELEMATICS Applications Program (TAP), Health Care Sector. In this paper we provide an overview of the system and the methodological approach adopted. Key characteristics with respect to the technical architecture and flexible customisation of different web and kiosk-based versions will be presented. In particular, the development of dedicated software for the procurement, structuring and management of the information knowledge-base is illustrated. Some of the most interesting findings from a cross-national study of 'health information needs on the internet' are presented along with information on the validation of the system by the general public, content providers and health care authorities.

## Introduction

In recent years, the number of people accessing the World Wide Web (WWW) has increased beyond expectation. All manner of users, from the casual surfer to the professional can avail themselves of this vast information resource, with health information and health promotion resources being prominent [1]. The Internet is seen as a new medium for the dissemination of health related information having the potential to reach a global audience. It is with this potential in mind that the idea of the European CATCH II (Citizens Advisory System based on Telematics for Communication and Health) project came about.

Funded by the European Commission under the Fourth Framework Research & Development TELEMATICS Applications Program (TAP) in the area of Health Care, the system is being developed by a consortium of partner organisations (universities, health care organisations and IT (Information Technology) companies) from Northern Ireland, Germany, Portugal and Italy. The aim of CATCH II, which has been in existence since January 1998, was to develop, evaluate, and disseminate a methodology and framework for the creation, maintenance, update, and configuration of multilingual, multimedia, medical information systems for the European citizen. The final objective is to provide a system that facilitates the collation, management and dissemination of a comprehensive health information knowledge-base that can be accessed easily via the Internet or stand alone kiosks. CATCH II is an extension of its predecessor CATCH I (an EU (European Union) project funded under the same program) to develop a touch-screen kiosk-based health information system. The key objective of CATCH II was the redesign and migration of the system from a stand alone kiosk version to a multi-functional kiosk and internet system.

Previous studies have demonstrated that much of the health information on the web is of variable and inconsistent quality, difficult to understand [2,3,4] and difficult for medical professionals to make direct contributions [5,6]. While the Internet has the capability of transforming the future of online health information systems, "...the potential for misinformation and confusion is there on the web" [7]. A significant pitfall with many systems is that they lack the endorsement of medical professionals and verification of the medical information provided. CATCH II attempts to address such problems by only offering material that has been created, proofed and certified by professionally recognised medical organisations, public health institutions and university faculties of medicine in several European countries. A user needs study clearly established the requirement to create easy to use tools for professional medical content providers, to give them direct interactive control of how information is input, managed and presented. This aspect is discussed further in the CATCH II methodology section.

## User needs study and technical/functional specification

Prior to the development of the system, a user needs study was conducted in the four participating countries. The study included two independent surveys - the first consisted of a survey of a stratified random sample of the *general public* and the second was a set of semi-structured interviews with a variety of *health care professionals* with a particular remit for health promotion. For the general public survey, respondents were asked for their opinions and attitudes towards a range of important issues such as health behaviour and lifestyle, attitudes to health promotion, familiarity with computing, the Internet and kiosk technologies and their use for health related information. Consumers involved were surveyed using a questionnaire based study in Germany (n = 300), Italy (100), Portugal (101) and Northern Ireland (122). A response rate of circa 90% was achieved in all countries. The objective was to identify any content and technical issues, including cross-cultural differences, which should be accommodated for in the development of a multi-lingual, multi-national health information system. It was also important to examine attitudes of the public about the future potential of kiosk and Internet-based health information systems. The semi-structured qualitative interviews with health care professionals were conducted among representatives from various organisations in Germany and Northern Ireland e.g. Federal Central for Health Education (BzgA) in Germany and the Western Area Health and Social Services Board in Northern Ireland. These organisations and their representatives were selected as key organisations within their respective national health care structures and the fact that 'health promotion' plays a large role in their day to day activities. In all, eighteen interviews were carried out in Germany and fourteen in Northern Ireland. All of the organisations asked to participate in the study did so willingly. The interviews were designed to elicit information on the various ways health promotion activities are managed and delivered within different European health care systems. Also, current methods of best practice in the creation, management and presentation of health promotion information were investigated. Respondents were asked their opinions about the use of the Internet and kiosks as modes for the delivery of health information and for ways in which our system methodology in particular could facilitate new and better ways for the creation, management and delivery of health promotion materials.

The results, summarised very briefly here, yielded some interesting findings with implications for the design of the functional and technical specifications of the system. From the general public survey, differences in health behaviour between countries were clearly evident. For example, people in the southern European countries are more likely to smoke while the northern Europeans are more likely to drink alcoholic beverages (especially beer and spirits rather than wine and in greater quantities). The implication is that a European health information system might require various languages and a different emphasis or approach to particular issues for particular countries. Other significant issues related to a general lack of satisfaction with current Internet and kiosk-based health information systems. In particular, respondents were less than complimentary about the volume and complicated (medical) nature of many texts and images, ease of navigation and general usability of some systems. Many were of the opinion that navigational tools should be made more intuitive to enhance the concept of a user-friendly system. Textual information and images were generally viewed as being too convoluted in terms of the medical language used. It was therefore in the remit of CATCH II to provide legitimate information in a format that is easy to interrogate and understand.

From the health care authority survey it was evident that the Internet and information communications technologies (ICT) in general have great potential for health promotion activities. To date though very few agencies were making use of such technologies, the Northern Ireland Health Promotion Agency (http://www.healthpromotionagency.org.uk/) and the German AOK health Insurance Company (http://www.aok-info.de/) being notable exceptions. It was also clear from the perspective of content creation, that the development of a simple user-friendly system for the transfer of existing materials and the creation and management of new materials into a suitable format for web and kiosk media would be extremely valuable. The point was regularly made that the owners and creators of health promotion information, being medical professionals and not computer scientists, would benefit greatly from such a method and that their needs should be taken into consideration.

**Figure 1:**
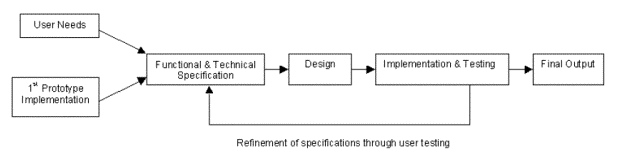
Iterative process of functional & technical specification

From a user needs study (of potential end users and content creators) and previous experience of the consortium team, the functional and technical specifications were identified to facilitate the successful implementation of the CATCH II system. The functional specification defined the performance requirements of the CATCH II system; the technical specification defined the technology that is required to meet the functional specification (Figure 1). Failure to meet the users' needs would result in the system failing to gain widespread support and acceptance. Both the user needs study and previous work on the prototype system provided input to these specifications to ensure the system was properly constructed.

## The CATCH II methodology

**Figure 2:**
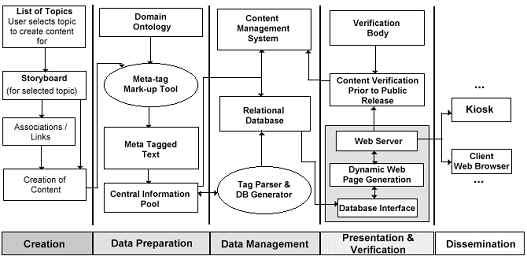
The CATCH II methodology

Taking into account the functional and technical specifications and the key findings of the user needs study, the CATCH II team developed a unique methodology that would act as the framework to carry our project through to implementation. The methodology itself is comprised of five main sections; content creation, data preparation, data management, verification and presentation and dissemination (Figure 2). The process begins with a content creator (e.g. a medical specialist) who has materials on a particular subject and wishes to incorporate them into the CATCH II system. The medical professional is core to this methodology; they are responsible for the provision of appropriate structured information and are placed at the beginning of the process in an attempt to provide freedom from excessive restrictions and regulations that may be imposed by technological factors [8,9,10]. All relevant materials for inclusion are 'storyboarded' to include all relevant links and associations between components (be they text, graphics, audio or video). The individual components are then incorporated into the system using one of two specially developed editing tools and an associated CATCH II medical domain ontology (as described later).

The texts created by the medical professionals are then structured and have metadata added in an XML (eXtensible Markup Language)-like format. With these tagged texts, the value added information allows alternative databases to be prepared for presentation use. The medical professional is responsible for the accurate content of the system along with the page authoring. As the system of tagging using the editing tools is very intuitive and easy to put into practice, the medical professional would have little problem in carrying out this procedure. Few computer skills are necessary to use the editors. As the system was a prototype methodology, medical experts involved in the project were eager to perform their role as content creators.

## CATCH II authoring environments

A key requirement identified from the user needs of the medical professional was the provision of an easy to use content authoring tool that would assist in the realisation of the domain ontology for the human body. The CATCH II team developed two individual tools, ***ConteXt*** and ***CEdit*** [11]. Both satisfied the methodology as outlined above but used different modes of delivery, i.e. ConteXt can be downloaded, installed on the client machine and be run locally whilst CEdit is a Java applet designed to run over the web. For the purposes of this paper the ConteXt editor is demonstrated.

The authoring environment is made up of two key components - an editor and a domain ontology, upon which the editors are built. An ontology defines the basic terms and relations comprising the vocabulary of a topic area, as well as the rules for combining terms and relationships to define extensions to that vocabulary. They are ' *explicit specifications of conceptualisations*'. The CATCH II ontology is not designed to become a scientific domain ontology like SNOMED [12] or MESH. What authors need to fulfil their task is a practical tool that allows them to characterise the nature and the intention of a text element.

***ConteXt*** is a tool that we designed for the structuring and management of reusable multimedia content. It grew out the need to help the medical professionals creating the content for websites [13,14,15,16]. ***ConteXt*** has the ability to structure, tag texts and then to store these texts in a central information pool. From this pool, the information can be extracted and displayed in a browser. Effectively, ***ConteXt*** is a text and graphic management tool with specialised functionality that is heavily based upon the domain ontology. The current ontology is comprised of medical terms specifically relating to the human body (cardiology and skin cancer). The tool allows editing of texts, addition of meta-tags and placement of texts into appropriate sections using the domain ontology. The author, or content creator, begins by creating a piece of text by directly typing new content or importing text for mark-up. This text must also have metadata added. Metadata is *data about data.* Its inclusion extends content reusability because it defines structural units and individual elements of the texts that can be manipulated and used in a variety of different contexts. Additional video and audio files can also be associated with the section of domain content.

**Figure 3:**
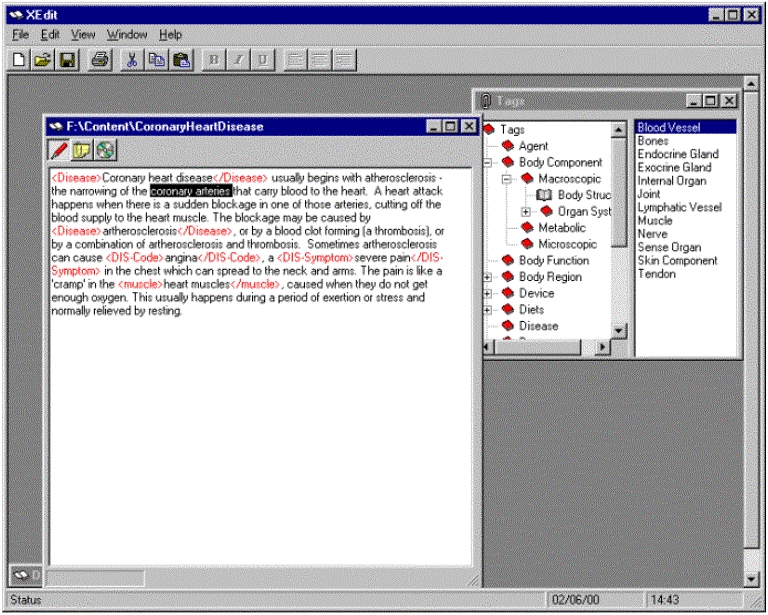
Example of using ConteXt to add metatags

One of the primary functions of ConteXt is to add metadata to created texts. The metadata is physically incorporated using XML(eXtensible Markup Language)-like tags [17,18]. These tags are defined by the domain ontology. To tag a piece of text, the user highlights the desired piece of text and then selects the appropriate tag from a menu. This process is similar, for example, to making a piece of text bold in a word-processor (see figure 3 above). A description of the ontology is dynamically loaded at runtime; this defines the tags available for selection by the content creator. Figure 3 shows how the tags can be selected from the 'Tags' window, which contains the entire ontology. Tagging texts in this manner is key to making the texts reusable. The tagging function maps directly to the underlying tables that warehouse the data.

The tagging process is particularly valuable when addressing the topic of information presentation. The tags allow the texts to be easily categorised and searched with a view to creating customised databases specifically for presentation purposes. For example, we can extract all texts which are connected with sunburn. These can then be placed in a simple database to be read for display by a kiosk or web application under that medical theme.

The current CATCH II Web site uses these procedures to dynamically produce HTML pages from the database. The web server accepts a request, queries the database, extracts the results and prepares a formatted web page that is then sent to the requesting client. The CATCH II web site was initially produced in English and German.

**Figure 4:**
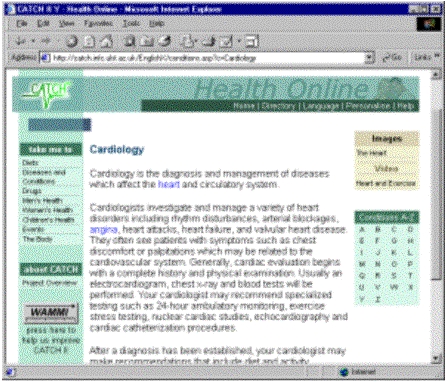
The web-based system

Kiosk-based systems have different technical considerations because of additional functional requirements [19,20,21,22]. In the case of CATCH II, the kiosk-based system should be functionally similar to the web-based system, and therefore should use similar underlying structures in an off-line fashion. That is, the server-side for the web-based system should be used as the framework to provide the requested information to the kiosk. This information can then be structured and presented in a format appropriate for touch screen kiosks. The CATCH II kiosk system deployed only a German translation. However, a direct translation of the skin cancer topics were replicated in the English website version. Figure 4 below shows a multimedia example of both the current CATCH web site and kiosk system.

**Figure 5:**
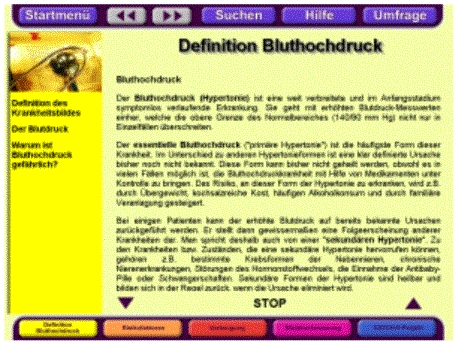
The kiosk-based system

## Validation

Throughout the validation phase of the project various procedures and de facto standard instruments were used to assess the value of key components within the overall project. In addition, workshops were set up to evaluate the responses of medical content providers and health promotion professionals. Validation was carried out from three very different perspectives; technological, end-user and health care authorities.

A standard industry tool, WAMMI (Web Site Analysis and MeasureMent Inventory), produced by the Human Factors Research Group in Cork, Ireland was adopted to evaluate the usefulness of the web system. It proved to be a convenient and cost-effective method of assessing the value and usefulness of the Internet system. WAMMI uses a scoring program with six categories: attractiveness, controllability, efficiency, helpfulness, learnability and global usability. Results showed that the web site scored above average on four of the five single measurement scales (except controllability). In particular, the two best scores were obtained for the attractiveness of the web site and the efficiency of the web site. The composite score measuring global usability revealed that overall the web site had been rated above average, indicating that users were generally happy with the function, style and feel of the site. A separate questionnaire was developed for the evaluation of the kiosk-based system. Respondents were confronted with different statements about the content, the presentation and the quality of use. Additionally, suggestions and opinions, which would help to design the kiosk system in a more interesting and informative way, were sought. On the whole, the participants of the validation assessed the kiosk version positively.

Validation of the editing software was performed with SUMI (Software Usability Measurement Inventory) developed by Nomos, Sweden. It is a rigorously tested and proven questionnaire-based method of measuring software quality from the end user's point of view. It is also considered as a consistent method for assessing the quality of use of a software product or prototype and can assist with the detection of usability flaws before a product is launched. Medical professionals, and other potential content creators, from the UK, Germany, Italy and Portugal were selected to use and experiment with the software and then complete a SUMI questionnaire.

The results indicated that respondents in general had very little difficulty learning to operate the software. They felt that it was efficient and they could perform data entry functions e.g. tagging of text and graphics easily. An interesting endorsement of the software was that many of the users would have recommended it to their colleagues, suggesting that it is a quality product.

Health care authority validation workshops were conducted in participating countries. The objective was to try and establish if those people directly involved in health promotion activities thought that the CATCH II system (its methodology and component parts) could play a realistic and practical role in the promotion of health within their respective domains and for the European citizen. From the feedback, it was very clear that all of the participants were impressed with the system and viewed it as a very innovative approach to providing health promotion information. It was also encouraging that many could see ways in which they could adapt CATCH II or components of the system into the practices of their own organizations.

A number of issues were consistently raised and discussed by participants. In general, they centered around the content of the system - for example, verification, certification, ownership and control of the information, quality assurance and consistency and the form in which information was presented to the public (i.e. the type of language used). This was an important finding as it highlighted some of the issues that the project was already trying to address directly. For example, the development of the remote editing tools was a deliberate attempt to give control of the creation, management and presentation of information to the content creators and health care specialists as opposed to technical professionals.

## Conclusions

The CATCH II project demonstrated the importance of engaging the user community of the intended target system in order to evolve a flexible methodology for its eventual realisation. This has been made possible by devoting the time to take into consideration the drawbacks of existing Internet-based health information systems, to consider comments from citizens in each country, and to engage the health authorities and medical professionals who could benefit from our approach. The methodology and tools we have developed under CATCH II can be easily customised to include other health modules, or indeed address other information-rich applications that require a presence on the Internet.

The development of the methods and processes described above will continue and attempt to embrace the advanced technologies of the Internet. We see our approach as having applications beyond the health domain and welcome the opportunity for further discussion and collaboration with colleagues and interested parties.
